# Daily patterns of loneliness and binge eating and food addiction using ecological momentary assessment

**DOI:** 10.1007/s10865-025-00575-w

**Published:** 2025-05-21

**Authors:** Lilia Margaryan, Kathryn E. Smith, Tyler B. Mason

**Affiliations:** 1 Department of Population and Public Health Sciences, University of Southern California, Los Angeles, CA USA; 2 Department of Psychiatry and Behavioral Sciences, University of Southern California, Los Angeles, CA USA

**Keywords:** Binge eating, Loneliness, Food addiction, Ecological momentary assessment

## Abstract

**Objective:**

Binge-spectrum eating disorders (BSEDs) are characterized by recurrent binge-eating episodes and have grown vastly in prevalence. Many individuals with BSEDs also report elevated food addiction (FA), which is described as a strong, irresistible urge to consume highly palatable processed food. Many studies have found individuals with BSEDs and/or FA often use food to soothe negative emotions–including loneliness, yet loneliness as a specific emotion associated with disordered eating is understudied. This study investigated trajectories of loneliness across the day and how loneliness trajectories were associated with daily binge-eating and FA symptoms using ecological momentary assessment (EMA).

**Methods:**

Adults with BSEDs and/or FA (*N* = 49; M_age_=34.9 ± 12.1; 77.1% cisgender female) completed an 11-day EMA protocol, which assessed loneliness, binge eating, and FA. Multilevel latent growth mixture models were used to empirically derive daily loneliness trajectories and evaluate associations with binge eating and FA.

**Results:**

Six daily trajectories of loneliness were found, which differed in intercept and slope of loneliness across the day. Compared to “stable low loneliness” days, “elevated early loneliness, decreasing then increasing” and “elevated early loneliness, decreasing” days showed higher daily FA symptoms. There were no significant differences between trajectories on daily binge-eating symptoms.

**Conclusions:**

The results support daily loneliness, particularly days with elevated early loneliness, as a salient factor associated with elevated daily FA symptoms. Thus, interventions targeting morning loneliness should be considered for FA intervention. There were several study limitations, such as inability to make causal conclusions, moderate between-subjects sample size, and lack of clinical interview assessment.

## Introduction

According to the Diagnostic and Statistical Manual of Mental Disorders Fifth Edition Text-Revised (DSM-5-TR), binge-spectrum eating disorders (EDs), including binge-eating disorder (BED) and bulimia nervosa (BN), are characterized by recurrent binge eating, which is defined as eating an amount of food that would be considered larger than what most people would eat in that specific period of time and a sense of lack of control over the eating episode (American Psychiatric Association, 2024). Food addiction is an overlapping but definitionally distinct condition, which is characterized by the overconsumption of ultra-processed foods alongside symptoms akin to substance use disorders, including continued use despite negative consequences, difficulties controlling consumption, and intense food cravings (Oliveira et al., 2021). Many individuals with binge-spectrum EDs also report elevated food addiction, which both involve strong urges to consume food and a feeling of loss of control over how much and what kind of food one is eating, despite awareness of potential health risks and consequences (e.g., obesity, diabetes, heart disease) (Di Giacomo et al., 2022). Loneliness has been shown to be associated with EDs and food addiction; however, research has been primarily cross-sectional (e.g., Dinçyurek et al., 2018; Kim et al., 2023; Pavlova et al., 2008; Tatsi et al., 2019). The goal of the current study was to increase empirical knowledge of loneliness as a contributor to EDs by examining associations between daily loneliness trajectories and binge-eating and food addiction symptoms.

## Significance of Binge-spectrum EDs and food addiction

Binge-spectrum EDs affect up to approximately 2.2% of the United States (U.S.) population, with BED affecting 3.5% of women and 2% of men and BN affecting 1.7-2.0% of U.S. women and 0.5–0.7% of U.S. men (Keski-Rahkonen, 2021). For food addiction, a meta-analysis of 53 studies across clinical and non-clinical samples showed an average prevalence of 20%, with the mean prevalence being 55% for those already diagnosed with BED and 48% for those with BN; some studies also reported higher prevalence in males compared to females (Praxedes et al., 2022). Binge-spectrum EDs and food addiction are associated with significant mental and physical health problems, including suicide risk, mood and substance use disorders, physiological complications and symptoms, and chronic disease (Brownley et al., 2016; Hudson et al., 2010; Keski-Rahkonen et al., 2021; Mitchell & Crow, 2009; Parylak et al., 2011; Rosten & Newton, 2019). Furthermore, data suggest that evidence-based interventions are only moderately effective for treating binge-spectrum EDs, with 50% of BED patients and 37% of BN patients being able to achieve abstinence after receiving treatment (Linardon et al., 2018). In addition, these EDs have significant relapse rates, with 10-year relapse rates being up to 30% for BED (Sala et al., 2023). Together the negative impact of binge-spectrum EDs along with the sub-optimal treatment outcomes requires new treatments, particularly ones that account for the contextual influences that predict binge eating.

### Associations of loneliness, binge eating, and food addiction

Humans, by nature, are social beings (Young, 2008). Hence, perceived lack of social support and connection have negative effects on an individual’s life, which often can manifest as mental health disorders, including EDs (Levine, 2012; Zhang & Dong, 2022). Specifically, loneliness involves subjective feelings of social isolation from others and differs from being alone in that people feeling lonely often want to connect with others (Rosenberg et al., 2013; Veronese et al., 2021). Numerous studies have found associations between loneliness and binge-spectrum EDs, binge-eating symptoms, and food addiction (Dinçyurek et al., 2018; Kim et al., 2023; Pavlova et al., 2008; Tatsi et al., 2019). Survey studies of non-clinical populations showed positive correlations between loneliness and greater food addiction symptoms (Dinçyurek et al., 2018; Tatsi et al., 2019). Consistently, using data from a U.S. population-based study, Kim et al. (2023) reported that adults with EDs (including BED and BN) had lower perceived social support compared to non-ED controls. Furthermore, in qualitative data from Czech women living abroad, loneliness was identified as a reason for engaging in binge eating (Pavlova et al., 2008).

**Mechanisms of loneliness and EDs and food addiction.** While studies have yet to determine the exact mechanisms of how loneliness may serve to increase risk of EDs and food addiction, several psychosocial and physiological pathways may link loneliness and binge eating and food addiction. Regarding psychosocial mechanisms, loneliness has been shown to be positively associated with emotion dysregulation, social withdrawal, and interpersonal difficulties, which are important risk factors for EDs (Heinrich & Gullone, 2006; Tatsi et al., 2019). Additionally, a meta-analysis outlined the physiological effects of loneliness, including increased risk of cardiovascular, immunological, and endocrine pathology (Holt-Lunstad et al., 2015). Physiological effects of loneliness, such as increased stress, increased hunger hormones (such as ghrelin, glucagon, and orexin), and other hormonal imbalances, may interrupt appropriate appetite and emotion regulation and in turn precipitate binge eating and food addiction symptoms (e.g., Culbert et al., 2016).

Moreover, a prominent model of EDs is the affect regulation model of binge eating, which suggests that increases in unpleasant affective states (e.g., guilt, sadness, stress, shame) drive binge eating as a way of regulating unpleasant negative emotions (Haedt Matt & Keel, 2011). Studies using ecological momentary assessment (EMA) have shown that negative affect increases and positive affect decreases prior to a binge-eating episode, and just after the episode, negative affect decreases and positive affect increases, acting as a negative reinforcement cycle for maintenance of the maladaptive coping behavior (Engel et al., 2016; Schaefer et al., 2020). Because loneliness is a unique emotion that specifically focuses on negative emotions related to unmet social needs (Yanguas et al., 2018), loneliness may be a specific emotion associated with binge eating and food addiction in line with the affect regulation model.

## Ecological momentary assessment (EMA) of loneliness and eating behaviors

EMA is a real-time data capture method that uses repeated measures to collect information from individuals throughout the course of their daily life (Trull & Ebner-Priemer, 2009). EMA methods overcome many limitations of standard survey-based assessments by allowing for the studying of within-day processes, decreasing the opportunity for recall bias and loss of variability from summary measures, and increasing generalizability to the real-world (Engel et al., 2016). Several EMA and daily diary studies have considered the role of loneliness in relation to binge eating. One study of college women using daily diary methodology found that greater daily loneliness was associated with higher daily binge eating, even after controlling for daily negative affect (Mason et al., 2016). However, loneliness and eating behaviors vary across the day and in different contexts and may not be accurately captured by retrospective questionnaires, including end-of-day measures. One recent study examined momentary loneliness using EMA in a sample of 483 food delivery application users and found that loneliness predicted greater body dissatisfaction but was unrelated to disordered eating urges (Portingale et al., 2023); although, this study did not examine associations between loneliness and disordered eating behaviors (like binge eating and food addiction symptoms). While urges often predict behaviors, urges do not always predict engagement in the associated behavior and sometimes people are not consciously aware of urges (Shaw, 2024), necessitating more research on state loneliness and eating behaviors.

### Daily loneliness trajectories and eating behaviors

Moving beyond traditional EMA analyses, some studies have used latent growth mixture models (LGMM) with EMA data to study daily trajectories in risk factors and how they are associated with eating behaviors. It is possible that risk factors (such as loneliness) may show distinct, prototypical trajectories across the day that associate differently with eating behaviors. Thus, rather than any one moment of high loneliness increasing risk for disordered eating, it may the case that a certain daily trajectory is associated with risk of disordered eating. In the context of the affect regulation model, two studies looked at daily trajectories of general negative affect and anxiety states and binge eating in women with EDs. Using EMA in a sample of women with BN, Crosby and colleagues (2009) identified nine trajectories of daily negative affect and found that binge eating was highest on days with stable high negative affect or increasing negative affect across the day compared to stable low negative affect. In a separate study of women with anorexia nervosa, binge eating was elevated on days with stable high, late increasing, and late decreasing anxiety trajectories compared to the stable low anxiety trajectory (Lavender et al., 2013).

While loneliness and general negative affect are associated, general negative affect measures do not fully capture the social dynamics inherent in the feeling of loneliness (Finley & Schafer, 2022; Yanguas et al., 2018). Furthermore, loneliness has been shown to vary within a day, which may be due to prior-day feelings of loneliness, physiological and biological processes (e.g., sleep, cortisol), and various contexts and social situations that occur across the day (Buecker et al., 2024; Doane & Adam, 2010; Johnson et al., 2024). Loneliness may fluctuate in ways differently from general negative affect, yet, to our knowledge, no prior studies have investigated the possibility of distinct trajectories of loneliness across the day nor how trajectories associate with binge eating and food addiction.

## The current study

While previous EMA research has studied momentary associations between loneliness and interpersonal problems and disordered eating cognitions and behaviors (Mason et al., 2021; Portingale et al., 2023), they have largely focused on general samples opposed to samples with EDs or food addiction. Also, to our knowledge, studies have yet to identify trajectories of loneliness and associations with behavioral outcomes such as binge eating and food addiction symptoms. In addition, prior EMA research on momentary loneliness and interpersonal problems focused on individuals with binge eating but less has focused specifically on individuals with BED and/or food addiction. Addressing these gaps, the present study recruited a clinically relevant sample and applied LGMM to identify distinct daily trajectories of momentary loneliness across an 11-day EMA protocol.

The objective of the current study was to use EMA to statistically define trajectories of loneliness across the day and examine how trajectories are associated with daily binge-eating and food addiction symptoms. While it is difficult to make specific hypotheses given the hypothesis-generating nature of the study, we hypothesized that loneliness would demonstrate different trajectories across the day and days characterized by elevated loneliness at some point during the day would be associated with higher binge eating and food addiction symptoms compared to days characterized by low loneliness.

## Methods

### Participants

Adults aged 18–64 with BED and/or food addiction (*N* = 49) were recruited into the Mobile Eating Activity and Lifestyle (MEAL) study (see Kalan et al., 2024). Participants were recruited from email lists from prior studies at the University of Southern California (Dunton et al., 2020; Ponnada et al., 2022; Wang et al., 2021) and also from Research Match, which is a National Institutes of Health platform in which volunteers across the United States register to be notified about research studies (Researchmatch.org). Participants completed a screening measure that assessed eligibility criteria. Inclusion criteria were: (1) meet criteria for BED and/or food addiction, (2) age 18–64, (3) read and speak English, (4) own and regularly use a smartphone, and (5) live in the U.S. Exclusion criteria were: (1) body mass index < 18.5 kg/m2, (2) self-reported severe intellectual disability, (3) current psychosis symptoms assessed with the Psychosis Screener (Degenhardt et al., 2005), (4) currently pregnant or breastfeeding, (5) inpatient or partial hospitalization in the past four weeks, and (6) previous bariatric surgery. Participants without BED, but with food addiction and another eating disorder (e.g., BN) were eligible. The Institutional Review Board of the University of Southern California approved the study procedures.

### Procedure

Eligible participants completed either online or Zoom informed consent. Enrolled participants were given a unique identifier, which was linked to all survey and mobile phone data. Then, online questionnaires and EMA instructions were given. Immediately following completion of baseline questionnaires, participants downloaded the Lifedata application (lifedatacorp.com) to their mobile phone and completed an 11-day EMA protocol. Each participant received an online document that provided instructions for downloading the application and completing the daily surveys; participants who completed the study via Zoom additionally received instructions from a trained research assistant. EMA studies in ED research have typically conducted EMA for 7–14 days (e.g., Lavender et al., 2013; Portingale et al., 2023); eleven days was chosen as a midpoint. Individuals who did not download the application and start EMA received reminders to do so. Because study enrollment was completed on a rolling basis, the 11 days were not the same for all participants. Lifedata maintains security and privacy safeguards, and data from Lifedata was linked to other study data through the unique identifier. The only identifying information collected through Lifedata were GPS coordinates, yet all participants were given the opportunity to opt out. At no point was EMA data linked to any other identifying information collected at baseline. EMA data were stored within the online LifeData platform, which is HIPAA compliant.

EMA was completed as participants went about their daily life and involved five semi-random signaled prompts as well as eating episode questions, which were participant initiated. EMA assessed various states and behaviors and were delivered between 8AM and 10PM in two-hour windows, with a prompt occurring randomly within each window. Prior research has used two-hour windows (Yang et al., 2020). Participants had an hour to complete the survey before it could no longer be accessed, which prevented backlogging of EMA reports. The mean prompt notification times were: Prompt 1: 9:26am (*SD* = 49 min); Prompt 2: 12:13pm (*SD* = 51 min); Prompt 3: 3:01pm (*SD* = 50 min); Prompt 4: 5:50pm (*SD* = 51 min); and Prompt 5: 8:36pm (*SD =* 49 min). In addition to semi-random prompts, participants were instructed to initiate surveys whenever they had an eating episode but were also able to input missed eating episodes during the semi-random prompts. Loneliness and food addiction were assessed through signal-contingent recordings, and binge-eating symptoms were assessed through eating episode recordings and signal-contingent recordings. Participants were compensated with up to $65, including $50 for completing the online survey and 11-day EMA protocol and an additional $15 for completing at least 80% of the EMA prompts.

## Measures

### Baseline measures

#### Demographics

Demographics and anthropometric information were assessed during the baseline questionnaire, including gender, age, years, height, weight, race/ethnicity. Self-reported weight and height were used to calculate BMI (kg/m^2^).

#### ED diagnoses

The Eating Disorder Diagnostic Scale DSM-5 version (EDDS DSM-5; Stice et al., 2017) assessed criteria for DSM-5 EDs using items that correspond to each DSM-5 ED criteria. The EDDS DSM-5 was used as a screener to measure if participants met criteria for BED as well as other EDs.

#### Food addiction symptoms

The Yale Food Addiction Scale 2.0 (YFAS 2.0; Gearhardt et al., 2022) was used to assess food addiction symptoms and clinically significant distress and impairment. Symptoms measured on the YFAS 2.0 aligned with the 11 DSM-5 substance use criteria. Items were dichotomized to denote meeting each criteria. A symptom count score was created, which is the sum of the 11 criterion and ranges from 0 to 11. Clinically significant food addiction was determined with symptom count and clinical significance scores and is defined as having both two or more symptoms and clinically significant distress.

### EMA measures

#### EMA loneliness

Current loneliness was assessed with a single item in which participants rated how lonely they currently felt on a scale ranging from 1 (*not at all*) to 5 (*extremely*). A similar item has been used in prior EMA research (Portingale et al., 2023).

#### EMA binge-eating symptoms

Participants rated binge-eating symptoms with four items. These included: “To what extent did you feel: (1) that you overate? (2) a sense of loss of control overeating? (3) driven to eat? (4) you could not stop eating?” A 5-point scale ranging from 1 (*not at all*) to 5 (*extremely*) was used. Using the same dataset as the current paper, psychometric analysis of these four items showed that they load onto a single-factor and show adequate reliability and concurrent validity (Mason et al., 2024). As such, we created a total binge-eating symptoms scores by taking the average of the four items.

#### EMA food addiction symptoms

Participants answered seven items on past two-hour food addiction symptoms, adapted from the Yale Food Addiction Scale 2.0. The items included: “My overeating got in the way of me doing the things I needed to do”; “I avoided work, school or social activities because I was afraid I would overeat there”; “My overeating affected my relationships”; “I tried and failed to cut down on or stop eating certain foods”; “I felt sluggish or tired from overeating”; “I was distracted by eating”; and “I felt distressed or had problems because of food and eating.” The response scale for each item was 1 (*not at all*) to 5 (*extremely*). This momentary food addiction scale showed adequate reliability and convergent validity (Varnado et al., 2024).

## Statistical analyses

Analyses were completed in SPSS version 29.0 (IBM; Armonk, NY) and Mplus version 8.3 (Muthén & Muthén, 2019). Descriptive statistics were calculated for demographics, clinical characteristics, and EMA. Multilevel models with loneliness as the outcome were run to examine differences in loneliness by demographics (i.e., age, BMI, gender identity, and race-ethnicity). For primary analyses, latent growth mixture modeling (LGMM) was utilized (Ram & Grimm, 2009), which allowed us to identify daily latent loneliness trajectories and to explore the association of the loneliness trajectories with daily binge eating and food addiction. LGMM assumes that data is composed of a collection of distinct subgroups, each with a prototypical growth curve or trajectory. The current analyses examined distinct trajectories of loneliness for each day across participants.

LGMM is a data-driven analysis, and multiple iterations were run to determine the best fitting model and number of classes to extract (Jung & Wickrama, 2008). The criteria for determining the best fitting model included model fit statistics: the log-likelihood, Akaike information criterion (AIC), Bayesian information criterion (BIC), sample size adjusted BIC (aBIC), and entropy. Lower values of all but entropy indicate better fit, and higher entropy indicates better classification accuracy. The best fitting models also considered the percentage of days belonging to each class; classes with low membership percentages may not be generalizable and have little power to test for group differences. Once the best fitting model was obtained, daily loneliness trajectories were plotted using the intercepts and linear and quadratic slope components. TYPE = COMPLEX MIXTURE was used to adjust the standard errors and fit statistics for clustering of days within participants (Clark et al., 2013). Loneliness trajectories were then compared on daily eating behaviors and baseline characteristics using Wald chi square tests.

## Results

### Demographics and clinical characteristics

The sample sociodemographics as well as ED and food addiction characteristics are presented in Table 1. Most participants demonstrated characteristics consistent with food addiction but not BED (*n* = 34), with 10 participants meeting criteria for BED but not food addiction and 5 participants meeting criteria for both food addiction and BED.

**Table 1:** Participant demographics and clinical characteristics, *N* = 49.

Variable	M (SD) or *n* (%)	Range
Demographics		
Age, years	34.9 (12.1)	20–64
BMI, kg/m^2^	33.7 (8.1)	18.6–53.7
Gender identity		
Cisgender female	37 (77.1%)	
Cisgender male	6 (12.2%)	
Transgender male	1 (2%)	
Gender questioning/non-conforming	3 (6.1%)	
Female sex at birth but gender identity was missing	1 (2%)	
Race-Ethnicity		
Non-Hispanic White	27 (55.1%)	
Hispanic	7 (14.3%)	
Black/African American	7 (14.3%)	
Asian	5 (10.2%)	
Multi-racial	3 (6.1%)	
Clinical Characteristics		
YFAS 2.0 symptom count^1^	6.88 (3.32)	0–11
EDDS probable diagnoses^2^		
Bulimia nervosa	25 (51.0%)	
Binge eating disorder	15 (30.6%)	
Subthreshold bulimia nervosa	3 (6.1%)	
Night eating syndrome	2 (4.1%)	
Atypical anorexia nervosa	2 (4.1%)	
No eating disorder	2 (4.1%)	

*Note*. BMI = body mass index; YFAS = Yale Food Addiction Scale; EDDS = Eating Disorder Diagnostic Scale

^1^Reflects the overall mean YFAS symptom count—the number of criteria met out of 11 (0–11)

^2^DSM-5 criteria were applied using responses to the EDDS

### EMA descriptives

Participants completed 2,555 EMA surveys, including 559 event-contingent and 1,996 signal-contingent surveys. The average EMA compliance was 75.3% to signal-contingent signals, and there were 1,358 eating episodes recorded across signals. Consistent with prior research (e.g., Naya et al., 2022), only days with at least three or more signals completed were included in analyses, leaving a total of 408 days. The descriptive statistics of study variable is displayed in Table 2. Analyses examining whether EMA reports of loneliness differed by demographic variables showed no differences in EMA loneliness by demographics (*p*s > 0.05).

**Table 2:** Descriptive statistics of study variables

Variable	*n*	M	SD
Daily binge eating	388	2.33	0.88
Daily food addiction	408	1.46	0.54
Loneliness EMA signal 1	359	2.01	1.27
Loneliness EMA signal 2	360	2.08	1.26
Loneliness EMA signal 3	360	2.13	1.31
Loneliness EMA signal 4	355	1.94	1.23
Loneliness EMA signal 5	343	1.93	1.23

### Main analyses

#### Identified loneliness trajectories

**Fig. 1:**
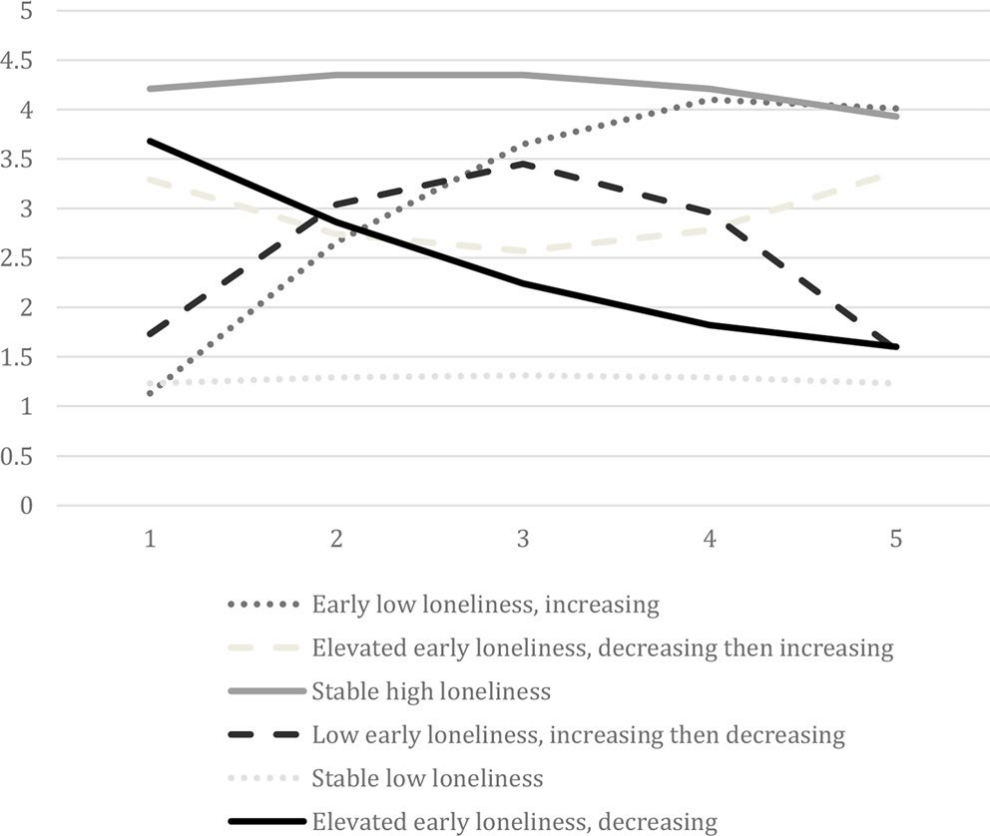
Six extracted trajectories of daily loneliness in 49 adults with binge spectrum eating disorders. Time 1 represents morning and Time 5 represents evening

The model fit statistics for solutions from 1-class (indicating loneliness varies the same across all days) to 7-classes (indicating seven distinct trajectories of loneliness across the sample of days) are shown in Table 3. Model fit continued to improve across all class solutions; however, class sizes decreased substantially at the 7-class solution, with one class having 6 days (1.5%). Thus, we settled on a 6-class solution to have more stable results and to examine group differences. Six distinct loneliness trajectories were identified across the 408 days; classes were named using the intercept and slope values (see Table 4). Figure 1 depicts class values on loneliness at EMA timepoints across the day for each trajectory. Most participants had all days fall into 1 (28.3%), 2 (23.9%), or 3 (28.3%) classes with a minority falling in 4 (15.2%), 5 (2.2%), or all 6 (2.2%) classes.

**Table 3:** Model fit statistics for latent growth mixture models

Number of Classes	LL	AIC	BIC	aBIC	Entropy
1	-2438.54	4895.07	4931.17	4902.61	-
2	-2372.76	4771.52	4823.67	4782.41	0.84
3	-2321.86	4677.73	4745.92	4691.98	0.89
4	-2265.31	4572.62	4656.86	4590.22	0.92
5	-2243.44	4536.88	4637.16	4557.83	0.91
6	-2212.66	4483.32	4599.65	4507.63	0.91
7	-2193.25	4452.50	4584.87	4480.16	0.91

*Note*. LL = log likelihood; AIC = Akaike information criteria; BIC = Bayesian information criteria; aBIC = adjusted Bayesian information criteria

**Table 4:** Mean intercept, linear slope, and quadratic slope levels of loneliness among different trajectories

Group	*n* (%)^1^	Intercept			Linear Slope			Quadratic Slope		
		M	SE	*p*	M	SE	*p*	M	SE	*p*
Group 1- Early low loneliness, increasing	15 (3.7)	1.13	0.09	< 0.001	1.80	0.34	< 0.001	-0.27	0.09	0.001
Group 2- Elevated early loneliness, decreasing then increasing	41 (10.0)	3.29	0.22	< 0.001	-0.74	0.23	0.001	0.19	0.07	0.005
Group 3- Stable high loneliness	44 (10.8)	4.21	0.19	< 0.001	0.21	0.19	0.270	-0.07	0.06	0.239
Group 4- Low early loneliness, increasing then decreasing	28 (6.9)	1.73	0.23	< 0.001	1.76	0.33	< 0.001	-0.45	0.09	< 0.001
Group 5- Stable low loneliness	246 (60.3)	1.23	0.04	< 0.001	0.08	0.05	0.079	-0.02	0.01	0.085
Group 6- Elevated early loneliness, decreasing	34 (8.3)	3.68	0.19	< 0.001	-0.92	0.19	< 0.001	0.10	0.04	0.017

*Note*. ^1^Percentages are out of the 408 classified days

#### Class 1

Class 1, labeled “Early low loneliness, increasing,” was characterized by low loneliness in the first EMA rating followed by increases in loneliness across the next prompts and accounted for 3.7% of the 408 classified days. There were 26.1% of participants who had at least one Class 1 day.

#### Class 2

Class 2, labeled “Elevated early loneliness, decreasing then increasing,” was characterized by elevated loneliness at the first rating that decreased toward the third prompt and then increased and accounted for 10.0% of the 408 classified days. There were 28.3% of participants who had at least one Class 2 day.

#### Class 3

Class 3, labeled “Stable high loneliness,” was characterized by high loneliness across the day and represents 10.8% of the 408 classified days. There were 78.3% of participants who had at least one Class 3 day. Demonstrated by non-significant linear and quadratic components, Class 3 had an overall high level of loneliness that did not change.

#### Class 4

Class 4, labeled “Low early loneliness, increasing then decreasing,” was characterized by low levels of loneliness at the first rating that increased during the middle of the day and decreased after and accounted for 6.9% of the 408 classified days. There were 65.2.% of participants who had at least one Class 4 day.

#### Class 5

Class 5, labeled “Stable low loneliness,” was characterized by low loneliness across the day and represents 60.3% of the 408 classified days. There were 89.1% of participants who had at least one Class 5 day. Demonstrated by non-significant linear and quadratic components, Class 5 had an overall low level of loneliness that did not change.

#### Class 6

Class 6, labeled “Elevated early loneliness, decreasing,” was characterized by elevated levels of loneliness during the first prompts that decreased across the subsequent EMA ratings and represented 8.3% of the 408 classified days. There were 45.7% of participants who had at least one Class 6 day.

#### Associations between classes and binge eating and food addiction

Wald chi-square tests showing differences between classes in binge eating and food addiction are displayed in Table 5. There were no significant differences between trajectories on daily binge-eating symptoms. There were significant differences for daily food addiction symptoms. Compared to “stable low loneliness” days, “elevated early loneliness, decreasing then increasing” and “elevated early loneliness, decreasing” showed higher daily food addiction symptoms. In relation to our hypothesis that days characterized by elevated loneliness at some point during the day would be associated with higher food addiction symptoms compared to days characterized by stable lower loneliness, our results showed that having elevated early loneliness appears to be most associated with higher daily food addiction symptoms.

**Table 5:** Comparisons between daily loneliness trajectory groups on binge eating and food addiction symptoms.

	Binge Eating			Food Addiction		
	M	SE	Χ^2^	M	SE	Χ^2^
Group 1- Early low loneliness, increasing	2.36	0.33	1 vs. 2 = 0.80, *p* =.371 1 vs. 3 = 0.37, *p* =.541 1 vs. 4 = 0.18, *p* =.676 1 vs. 5 = 0.27, *p* =.607 1 vs. 6 = 0.12, *p* =.726	1.57	0.18	1 vs. 2 = 3.07, *p* =.080 1 vs. 3 = 0.03, *p* =.874 1 vs. 4 = 0.15, *p* =.703 1 vs. 5 = 1.73, *p* =.188 1 vs. 6 = 0.04, *p* =.851
Group 2- Elevated early loneliness, decreasing then increasing	2.72	0.31	2 vs. 3 = 0.35, *p* =.554 2 vs. 4 = 0.26, *p* =.608 2 vs. 5 = 2.61, *p* =.106 2 vs. 6 = 0.57, *p* =.449	1.93	0.21	2 vs. 3 = 2.17, *p* =.141 2 vs. 4 = 1.73, *p* =.188 **2 vs. 5 = 7.91,** ***p*** **=.005** 2 vs. 6 = 2.86, *p* =.091
Group 3- Stable high loneliness	2.50	0.23	3 vs. 4 = 0.02, *p* =.934 3 vs. 5 = 1.43, *p* =.232 3 vs. 6 = 0.001, *p* =.973	1.53	0.17	3 vs. 4 = 0.21, *p* =.644 3 vs. 5 = 1.54, *p* =.215 3 vs. 6 = 0.11, *p* =.740
Group 4- Low early loneliness, increasing then decreasing	2.53	0.24	4 vs. 5 = 1.79, *p* =.181 4 vs. 6 = 0.02, *p* =.888	1.65	0.21	4 vs. 5 = 2.50, *p* =.114 4 vs. 6 = 0.07, *p* =.793
Group 5- Stable low loneliness	2.19	0.13	5 vs. 6 = 2.88, *p* =.089	1.32	0.06	**5 vs. 6 = 4.13**,***p* =.042**
Group 6- Elevated early loneliness, decreasing	2.49	0.21	-	1.61	0.15	-

#### Associations between classes and baseline demographics and clinical characteristics

For age, “stable low loneliness” days were associated with older age (*M* = 35.95) compared to “low early loneliness, increasing then decreasing” (*M* = 29.00; *Χ*^*2*^ = 6.17, *p* =.013) and “elevated early loneliness, decreasing” (*M* = 29.97; *Χ*^*2*^ = 7.39, *p* =.007) days. For baseline food addiction symptom count, “stable low loneliness” days were associated with lower baseline food addiction symptom count (*M* = 5.84) compared to “elevated early loneliness, decreasing then increasing” (*M* = 7.73; *Χ*^*2*^ = 4.24, *p* =.040) and “stable high loneliness” (*M* = 8.24; *Χ*^*2*^ = 4.08, *p* =.043) days. For baseline binge-eating frequency, “stable high loneliness” days were associated with higher baseline binge-eating frequency (*M* = 14.79) compared to all other days (all *p*s < 0.001), and “stable low loneliness” days were related to lower baseline binge-eating frequency compared to “elevated early loneliness, decreasing then increasing” (*M* = 9.36; *Χ*^*2*^ = 5.44, *p* =.020) days. There were no differences with BMI.

## Discussion

It is known that loneliness is associated with binge-spectrum EDs and food addiction, yet most research to date has been cross-sectional in nature (e.g., Pavlova et al., 2008; Tatsi et al., 2019). Using a similar EMA protocol to past research (Mason et al., 2021; Portingale et al., 2023), the present study identified daily patterns of loneliness in adults with binge-spectrum EDs and/or food addiction and examined associations between loneliness trajectories and daily binge eating and food addiction symptoms.

The findings of this study supported the hypothesis that days marked by elevated loneliness levels throughout the day were associated with increased food addiction symptoms, yet it was not the days marked by the stable high levels of loneliness that had the greatest food addiction. Rather, days characterized by elevated morning loneliness–either slightly decreasing across the day or decreasing early and then rising again–were associated with higher daily food addiction symptoms, suggesting a potential role of periods of elevated loneliness in the morning as a key intervention period in treating food addiction symptoms. One mechanism by which these specific trajectories may be associated with elevated daily food addiction symptoms is reduced capacity for self-regulation or as a coping mechanism, which may impact ability to resist cravings and urges to eat. In contrast to the high stable group, it may be the case that elevated loneliness in the morning that later fluctuates throughout the day indicates individuals engage in maladaptive behaviors (such as overeating) to cope; however, more research will be needed to determine this. This is consistent with the interpersonal model of EDs, which suggests that social difficulties (such as loneliness) precipitate increases in negative affect and in turn disordered eating behaviors (Rieger et al., 2010). In addition, momentary food addiction symptoms included failed attempts to restrict food intake; it may be that distress associated with elevated morning loneliness combined with attempts to control food intake (which often begin at the start of the day) increase susceptibility to overeating across the day, as posited by Loth and colleagues’ (2016) self-control model.

Morning loneliness that remains somewhat elevated may also impact biological stress processes (like cortisol) and psychological emotion and stress regulation processes (Doane & Adam, 2010). According to Social Safety Theory, social connection is fundamental to human’s wellness and social threats (including loneliness) can activate a maladaptive biological stress response (Slavich, 2022). In line with this theory, daily loneliness has been shown to alter the diurnal cortisol slope and the cortisol awakening response, which are indicators of stress (Miller et al., 2007; Stalder et al., 2025). Research has found that higher loneliness in the prior day (which may relate to morning loneliness) was significantly associated with an increased cortisol awakening response the following morning (Doane & Adam, 2010).

Increased cortisol awakening response and diurnal cortisol slope could increase stress, negative affect, and cravings (Epel et al., 2001), which may potentiate food addiction symptoms. Chronic elevations in cortisol have been shown to increase food intake, in part by downregulating insulin and leptin—two hormones that typically act to reduce cortisol levels during acute stress (Adam & Epel, 2007). However, when cortisol remains elevated, this regulatory balance is disrupted, promoting greater food consumption. Additionally, cortisol has been shown to enhance the perceived reward value of food, making the reward pathway of the brain more sensitized to highly palatable foods, such as those high in saturated fats and carbohydrates (Adam & Epel, 2007). This activation of the reward system releases neurotransmitters such as dopamine and endogenous opioids, which may contribute to stress-induced cravings and food addiction symptoms (Römer et al., 2023). There are nuanced associations between loneliness, sleep, and stress (both psychological stress and cortisol) (O’Connor & Rogerson, 2024; Okamura et al., 2011), which may interact to bring on food addiction symptoms. For example, poor sleep may interact with morning loneliness leading to a maladaptive cortisol response, which in turn can increase appetite, negative emotions, and reward processes that ultimately precipitate food addiction symptom severity.

The analyses did not support the hypothesis that loneliness trajectories would be associated with daily binge-eating symptoms, as no statistically significant differences were observed. According to the interpersonal model of binge eating, elevated negative affect acts as a mediator variable between interpersonal difficulties and binge eating (e.g., Karam et al., 2020). Prior research has also found the momentary negative affect and not loneliness was related to disordered eating urges (Portingale et al., 2023), suggesting that models of loneliness and binge-eating should account for the more general role of negative affect. In addition, a single loneliness item may not fully capture the loneliness construct and its relationship with binge eating. For instance, a previous daily diary study using multiple daily items to measure loneliness showed a significant association (Mason et al., 2016).

LGMMs were not able to establish a causal association between loneliness trajectories and eating behaviors as we were not able to examine temporal ordering between loneliness and binge-eating or food addiction symptoms. For example, it may be that loneliness fluctuated around binge-eating and food addiction symptoms, which would be consistent with affect regulation models (e.g., Schaefer et al., 2020). As such, engaging in binge eating or overeating could have directly lowered feelings of loneliness. Furthermore, some of the food addiction items included interpersonal aspects (e.g., overeating affected relationships), which may be tied to reports of loneliness. Altogether, this suggests a complex association between loneliness, emotion regulation, and eating behaviors, which include bi-directional effects and feedback loops.

Regarding associations between loneliness trajectories and baseline characteristics, younger participants were more likely to report several trajectories marked by elevated periods of loneliness compared to older participants. This finding is consistent with previous research, from a 2018 national survey among adults in the U.S. showing that loneliness decreases with age (Bruce et al., 2019). Though loneliness trajectories were not significantly related to daily binge eating, there were statistically significant differences with baseline binge eating. Individuals with greater baseline binge-eating frequency or food addiction symptoms were more likely to have trajectories characterized by higher loneliness (e.g., “stable high loneliness” and “elevated early loneliness, decreasing then increasing”). This finding is consistent with the known cross-sectional association between high loneliness and binge eating and food addiction (e.g., Mason, 2024; Tatsi et al., 2019).

### Theoretical and clinical implications

Our findings support interpersonal theories for food addiction symptoms and provide novel contextual information about how the nature of loneliness across the day may associate with food addiction symptoms. Results suggest that the interpersonal model may be adapted to support the role of early loneliness in the day as a risk factor for food addiction. However, since significant results were not seen for loneliness trajectories and daily binge eating, a contextually adapted model of loneliness may not be as applicable to binge eating. However, analyses do suggest the relevance of interpersonal models to binge eating, given associations between baseline binge-eating frequency and loneliness trajectories marked by periods of high loneliness. Overall, prior EMA and daily diary research (Mason et al., 2016; Portingale et al., 2023) and the current results show mixed evidence for associations between daily and within-day loneliness and binge-eating symptoms, which establishes a need for more understanding of how daily and within-day loneliness are related to binge-eating symptoms so theoretical models can be developed. Yet, significant associations between certain loneliness trajectories and daily food addiction may suggest that loneliness has a stronger association with the psychological experience surrounding overeating (e.g., impaired control, psychosocial impairment) compared to behavioral symptoms.

According to our study results, daily feelings of loneliness may be a useful target for interventions focused on reducing food addiction symptoms, particularly interventions that address morning loneliness. Several prolonged loneliness interventions have already been successfully identified and tested. Some examples include social skills training, mindfulness meditation, increasing social support, volunteering, group exercise, and structured therapy (Hoang et al., 2022; Veronese et al., 2021). However, fewer interventions have specifically focused on targeting real-world, acute loneliness, yet our study demonstrates that interventions targeting momentary and daily loneliness may be important for the treatment of adults with food addiction symptoms. Ecological momentary interventions (EMIs) are interventions delivered to people in real-time, most often through smartphone apps (Smith & Juarascio, 2019) and have shown preliminary efficacy for targeting binge-spectrum EDs (Juarascio et al., 2023; Kim et al., 2023; Shingleton & Palfai, 2016). Specifically, just-in-time-adaptive-interventions (JITAIs) may be useful for providing momentary interventions at critical periods of risk for an individual (Nahum-Shani et al., 2018), such as when loneliness is high. JITAIs are designed to provide personally tailored support to an individual in real time, based off their psychological and contextual states (Nahum-Shani et al., 2018). Given identification of elevated morning loneliness as a risky period for daily food addiction symptoms, JITAIs could provide strategies for combatting loneliness in the morning to reduce daily food addiction symptoms. For example, participants could be given intervention techniques in the morning, such as encouraging social interactions (e.g., interactions with family and friends) as well as planning and participating in in-person activities, which have all been shown to associated with lower momentary loneliness ratings (Zhaoyang et al., 2022). Ultimately, further research will be needed to determine the feasibility and efficacy of various loneliness interventions for EDs and food addiction, including the role of individual differences (e.g., personality, ED severity) in moderating the feasibility and efficacy of interventions.

### Limitations

Some limitations of this study should be acknowledged. One of the main limitations is the inability for the LGMM analysis to establish a causal association between loneliness trajectories and eating behaviors as we were not able to examine temporal ordering between loneliness and binge-eating or food addiction symptoms. Next, the between-subjects sample size of the study was relatively small, which may limit the generalizability of the findings to large-scale populations, including across different cultures and sociodemographic groups. People from collectivist cultures – such as those in East Asia, tend to have stronger social environments and place a greater emphasis on community and relationships, which could act as a protective factor against feelings of loneliness (Barreto et al., 2021). Meanwhile, people from individualistic cultures such as those in Western Europe and North America tend to place greater emphasis on themselves, and on average, have weaker social ties and less social support, which puts them at a higher risk of experiencing loneliness (Barreto et al., 2021). Though there was racial-ethnic variation in our sample, all participants were from the U.S., an individualistic country, which limits our ability to generalize the data obtained on a global level. Because individuals with BED and food addiction generally show greater loneliness compared to those with less symptomatology, our results may not generalize to people without ED symptoms or lower levels of symptoms as they may have less periods of high loneliness or loneliness that fluctuates in different ways. Relatedly, given the smaller sample size, not all participants had days falling into each class, and some participants may have driven identification of certain classes. In addition, given the small sample size, covariates were not included. Finally, the small sample size may have led to a decrease in the statistical power of our study, which has been shown to increase the likelihood of a type II error (a false negative), which could have occurred when comparing loneliness trajectories and binge-eating symptoms (Shreffler & Huecker, 2023).

Although overall compliance was acceptable, some participants did not complete all the prompts, which may lead to reporting biases. The average EMA compliance was 75.3% to signal-contingent signals. Albeit slightly lower, our compliance rates were in line with compliance rates found in a meta-analysis of 477 EMA studies (i.e., 79%; Wrzus & Neubauerthe, 2023). The meta-analysis showed that studies with financial incentives had higher compliance. Use of incentives in our study may have helped increase our compliance rate. A separate meta-analysis by Williams and colleagues (2021) found that prompt frequency per day and the number of items per prompt were significantly related to compliance. Our compliance may have been lower compared to some other studies because we had two prompt types (event- and signal-contingent) and included numerous items (to increase breadth of assessment). Some research has found that prompts with greater negative affect and lower positive affect (which are related to loneliness) predict non-compliance (Murray et al., 2023; Williams-Kerver et al., 2021), suggesting that prompts with higher loneliness may have been missed.

While we had 408 days of data, it is possible that a larger sample of days would have elucidated more daily trajectories of loneliness. Consistently, in the LGMM, model fit statistics continuously improved with the addition of more classes, but the sample size did not allow for more trajectories to be extracted. This could be achieved by recruiting more participants or increasing the number of days of EMA. Increasing the number of days would heighten participant burden, which would necessitate providing greater compensation to obtain adequate compliance. All data were collected with self-report measures. As such, ED diagnoses and food addiction symptoms were not verified with clinical interviews. There may have been recall biases, including under- or over-reporting of symptoms. As such, study findings may not generalize to samples with clinician and/or interview assessment of ED and food addiction symptoms. Further research would benefit from a larger, verified ED sample to draw more conclusive findings regarding the associations between daily loneliness and eating behaviors in BED and food addiction. Additionally, more DSM-tied measurement of state constructs is needed in future research.

## Conclusions

In summary, the findings of the current study showed associations between loneliness trajectories and daily food addiction symptoms but not daily binge eating. Mechanisms of the association between elevated morning, decreasing loneliness and elevated morning, decreasing then increasing loneliness and food addiction should be investigated in future research. Future observational research should further probe the association between morning loneliness and food addiction symptoms, including biopsychological mechanisms (e.g., stress, cortisol, sleep), using multi-method assessment (e.g., pairing EMA with actigraphy and saliva sampling). Integrating daily interventions for loneliness into treatment for food addiction symptoms may lead to better treatment outcomes. Specifically, results suggest interventions beginning in the morning may show the greatest efficacy. Next steps include studies testing ecological momentary interventions that provide loneliness interventions (e.g., encouraging social support, mindfulness) in the morning to gather empirical support for the efficacy of these interventions. In addition, these studies should examine potential moderators of intervention efficacy (e.g., personality, ED symptoms) to understand who benefits from these types of interventions.

## Data Availability

Data is available by request from the authors.

## Code availability

Code is available by request from the authors.
